# Introductory Address on Anatomy

**Published:** 1860-02

**Authors:** 


### BOOK AND PAMPHLET NOTICES.

Introductory Address on Anatomy. By Titus Deville, M. D, Prof. of Anatomy in Lind University, Chicago.

This address will commend itself for its originality, and the vigor and terseness of style in which the thoughts are expressed. The author has shown himself not only in possession of great mental vigor, but of habits of close thought and observation. Prof. Deville is not one of those who believes that knowledge comes by intuition, but like some others, his department has been and still is the grand ruling and controlling motive in life. To a thorough knowledge of his subject he unites a happy tact in imparting and impressing what he wishes to teach upon the mind of his student. Chaste and animated in his style, he fixes the attention of his class, and gives full assurance of his real worth as a lecturer. It is not the information that is conveyed, nor the polished and agreeable form in which the matter is presented, that renders addresses of this nature so eminently attractive; but the powerful stimulus such appeals inevitably give to the pursuit of knowledge in the minds of those to whom they are specially addressed. The following extracts from the *brochure* itself, will more fully acquaint the reader with its scope and character.

"On the faithfulness and excellence of anatomical details, blended with a highly cultivated taste, does the celebrity of the works of the sculptor and life-painter depend, and none have earned for themselves an undying reputation who have disregarded the study of anatomy as essential to that perfection which confers the stamp of genius on their productions. Whether we inspect the marvellous skill displayed in the Venus de Medici, Diana, Apollo Belvidere, Laocoon, Elgin Marbles, and other relics of classic art, or look to the period of the revival of art, at the works of a Michael Angelo, and a Leonardo da Vinci, down to the Etty of our own times, all evince that careful and correct appreciation of form and outline which can only be acquired by the teachings of *anatomy.* But of all the sciences which anatomy enriches, and to which she contributes her aid, none have received such a solid and brilliant impetus as Geology. The scroll of time has been read in the rocks and caverns of the earth, the matters that form the earth's exterior covering, have been found not to be a rude and confused mass, but a systematic arrangement, having a relative position and structure, and containing fossil remains of animal and vegetable life, differing in form and species, and belonging to another order of things than that which now surrounds us. These remains, detected by the sagacity of a Cuvier, and completed by Agassiz, Grant, Forbes, Owen, and others, have become the interpreters of the world's primeval history, and disclosed the fact that animal and vegetable forms, unlike anything now to be found on the earth, have lived in ages long, long ago, anterior not only to human records, but to the existence of man himself. They have disappeared by ordained convulsions of nature; whose history these hitherto silent oracles have at length disclosed, and have been succeeded by others of a different organization, and living under different physical conditions, which in turn have given place to the existing world of life, of which man, earth's thoughtful lord, is the crowning work. * * * *

The characteristics which distinguish the workmanship of nature in the construction of the skeleton, and excite a discriminative and judicious admiration, are found in the rigid economy conspicuous in every part, the diversified application of each single contrivance, the effective employment of apparently insignificant advantages—the accurate adjustment of the capabilities of each organ to the special function which it is designed to perform, and the particular strain which it has to support—the *elasticity* of one, the *rigidity* of another, the *tenuity* of a third, the *density* of a fourth; and the wonderful combination of lightness and durability which results to the fabric, considered as a whole.

What a remarkable piece of mechanism is the vertebral column!

Strong enough to support several hundred weight, yet pliant and elastic; furnished with levers and muscles, by which it is bent in every direction, yet lodging an organ susceptible of injury from the slightest pressure; formed, for lightness, of a loose and reticular tissue, yet capable of sustaining without fracture, shocks, strains, and contortions of considerable violence: this column certainly combines the most opposite qualities, and performs functions apparently incompatible. * * * * *

The functions of the hand, though for the most part matters of common experience, assume a new interest when considered in connection with its anatomical structure. The number and variety of its functions contrasted with the simplicity of the mechanism by which they are performed, illustrate the characteristic tendency of nature to produce, by the simplest possible means, the most numerous and diversified possible results. Guided by this general truth, let us review some of the ordinary actions of the hand. Compare, for instance, the light and gently varied compression with which it confines a fluttering bird, to the firm and unrelaxing hold with which it grasps a warlike weapon, or wields some heavy tool. Consider the swiftness of its movements in following the speaker with the pen; their variety in loosening a tangled knot; their nicety and precision in passing a thread through the eye of a needle. How steadily it guides the edge of the scalpel in a critical operation of surgery; with what singular truth it shapes the course of the school boy's marble, or adjusts his arrow to its mark! Nor are these the most wonderful of its performances. Trained to the juggler's sleight, its joints become yet nimbler and more pliant. Its evolutions, in the practice of several mechanical arts, are swifter than the eye can follow, of unerring regularity, independent of the guidance of vision, and productive of the most surprising results. In the musician, the sculptor, and the painter, it becomes the minister of more subtle volitions, and a higher instinct; in them accordingly, it requires still greater freedom and fluence of motion, a yet more exquisite refinement and fidelity of touch. In the orator it assumes a new character, and functions of an entirely different order. For him it is a powerful organ of expression, an indispensable auxiliary to speech. Accompanying with significant gestures the thoughts and emotions of the mind, it becomes the visible exponent of its secret workings; *the tongue*, so to speak, *of a language common* to all mankind. Bring together the wandering Arab—the red warrior of the American forests—the feathered barbarian of Africa—the civilized European. Which of them will mistake the meaning of a hand clenched in anger, or shaken in defiance; stretched abroad in the attitude of command, or raised to heaven in solemn attestation; waved triumphantly above the head, or pointing the finger of scorn; beckoning to summon attendance; barring the lips to enjoin silence; calmly extended in benediction; flung widely forth in dispair; covering the face in shame; wrung in the bitterness of grief; spread and shuddering in horror; or folded tranquilly in prayer? This delicate organ, capable as we have seen, of moving with the speed and precision of clockwork, may be doubled to form a weapon of offence, and employed, in the manner of a bludgeon, to give heavy blows, or to repel the strokes of an assailant. These violent concussions it sustains uninjured; eluding its force by their elasticity; and returning with unimpaired activity to the operations of the lathe or the loom.

When we consider the universality of the adaptation of means to ends—so constant that it cannot be the effect of chance—and the consummate harmony of the whole result so immeasurably transcending the highest efforts of human genius, it seems scarcely possible to arrive at any other rational conclusion, than that the universe, with all it contains, is the work of one Almighty and Benevolent Mind. The philosopher who has attained the highest summit of mortal wisdom, is he, who if he use his faculties aright, has the clearest preception of the limits of human knowledge, and the most earnest desire for the lifting of that veil which separates him from the Unseen. He then has the strongest motives for that humility of spirit and purity of heart, without which, we are assured, none shall meet their heavenly reward.

There is in medical science still, that dark centre which none of us have been permitted to penetrate; but it is surrounded by numerous investigators which give it now a hopeful light. From that brilliant circle, let it be your ambition each to snatch a burning brand, and penetrating the darkest recesses of the shade, there deposit your contribution of love. And, let us hope, that in no distant day there may arise some mighty genius who will gather up those scintillations, and combining them into one vast torch of truth, elevate it far above the obstructions of ignorance and folly, where it may burn with an unbroken lustre, and penetrate the remotest corner of the gloom!"

